# Effect of Hydration in Corona Layer on Structural Change of Thermo-Responsive Polymer Micelles

**DOI:** 10.3390/polym11020382

**Published:** 2019-02-22

**Authors:** Yusuke Akino, Kosuke Morimoto, Kengo Tsuboi, Satoshi Kanazawa, Isamu Akiba

**Affiliations:** Department of Chemistry and Biochemistry, The University of Kitakyushu, 1-1 Hibikino, Wakamatsu, Kitakyushu 8080135, Japan; ism94@mac.com (Y.A.); y7maa013@eng.kitakyu-u.ac.jp (K.M.); y7maa007@eng.kitakyu-u.ac.jp (K.T.); w5maa003@eng.kitakyu-u.ac.jp (S.K.)

**Keywords:** small-angle X-ray scattering, polymer micelle, temperature responsiveness, hydration

## Abstract

The effect of hydration in corona layer on temperature responsiveness of polymer micelles consisting of poly(*N*-vinyl pyrrolidone)-*block*-poly(*n*-octadecyl acrylate) (PVP-*b*-PODA) was investigated. Small-angle X-ray scattering and dynamic light scattering showed two-step shape change of PVP-*b*-PODA micelles around 45 and 65 °C with elevating temperature, although only one-step shape change was observed at 45 °C in cooling process. In the first step, shape of PVP-*b*-PODA micelles was changed from disk to ellipsoidal oblate at the melting temperature (*T*_m_) of PODA, although similar micelles consisting of another amphiphilic block copolymers containing PODA simply changed from disk to sphere at the *T*_m_ with elevating temperature. PVP-*b*-PODA micelles changed to spherical shape above 65 °C. Two-dimensional (2D) ^1^H-NMR showed the PVP chains were perfectly dehydrated above 65 °C. Therefore, it was suggested that the appearance of ellipsoidal shape between *T*_m_ of PODA and 65 °C was caused owing to shape memory effect of pseudo network of corona layer due to robust hydration of PVP chains.

## 1. Introduction

When amphiphilic block copolymers are dissolved in aqueous solution, they undergo self-assembly into polymer micelles consisting of hydrophobic core and hydrated corona [1]. The polymer micelles have been expected to be drug carriers in drug delivery system (DDS) because they can uptake hydrophobic drug compounds in their hydrophobic cores in aqueous solution [2,3,4]. For the DDS particles, stable retention of drug compounds is the critical issue to reduce side effects [5]. In order to control these properties, stimuli-responsive polymer micelles have been attracted much attention [6,7,8].

As means for introducing stimuli-responsiveness to polymer micelles, it is preferable to use a water-soluble polymer, which undergoes phase separation with water triggered by pH or temperature change, such as poly(*N*-isopropyl acrylamide) [6,7,8] or a crystalline hydrophobic polymer [9,10,11]. Among them, we focus on introducing a crystalline hydrophobic polymer as a thermos-responsive polymer because melting-crystallization transition should give sharp and large volume change at the melting temperature. Especially, a hydrophobic polymer having octadecyl group as a crystalline component is a good candidate for DDS particles because melting temperature is close to temperature of inflammation, which is higher than general body temperature. When a crystalline hydrophobic polymer is introduced to a polymer micelle, the shape of polymer micelles should be changed at the melting temperature (*T*_m_) due to a large change in volume fraction of hydrophobic core. Generally, the shape of a polymer micelle containing a hydrophobic crystalline polymer should be changed from disk to sphere at melting temperature in heating process [12]. However, if robust hydration is formed in a corona layer of polymer micelle, its effect on shape change of polymer micelle induced by melting in hydrophobic core should not be negligible. Although the importance of hydration in water-soluble polymers has been recognized, its effect on shape of polymer micelles has not been well investigated. It has been well known that poly(*N*-vinyl pyrrolidone) (PVP) is strongly hydrated in aqueous solution [13,14]. Therefore, it is expected that the influence of hydration in corona layer appears in the temperature responsiveness of polymer micelles consisting of PVP as a hydrophilic polymer and a crystalline hydrophobic polymer. Thus, the aims of this study were to investigate effect of hydration in corona layer on temperature responsiveness of polymer micelles consisting of PVP-*block*-poly(*n*-octadecyl acrylate) (PVP-*b*-PODA).

## 2. Materials and Methods

Reagents: *n*-Octadecylacrylate (ODA), *N*-vinyl-2-pyrrolidone (VP), 1-dodecanethiol, an tetraoctylmethylammonium chloride were purchased from Tokyo Chemical Industry Co Ltd. (Tokyo, Japan). 4,4′-Azobiscyanovaleic acid (ACVA) acetone, NaOH, and activated alumina were purchased from Wako Pure Chemicals (Tokyo, Japan). ODA and VP were treated with aqueous NaOH solution (5 wt %) and activated alumina, respectively, before used to remove inhibitors. The other reagents were used as obtained.

Synthesis of chain transfer agent (CTA): CTA used for reversible addition fragmentation transfer radical polymerization (RAFT) was synthesized following the method described literature [15]. 1-Dodecanethiol (10.4 mL, 60.0 mmol), tetraoctylammonium chloride (0.974 g, 2.40 mmol), and acetone (28.9 g, 497 mmol) were mixed in a three-necked round-bottom flask under dry N_2_ atmosphere. The flask was cooled to 10 C. Then, aqueous NaOH solution (50%) was added dropwise to the flask for more than 20 min, and stirred for 15 min.

**Scheme 1:**
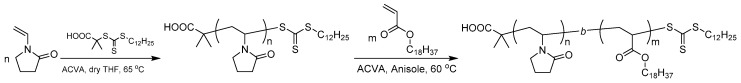
Synthesis of poly(*N*-vinyl pyrrolidone)-*block*-poly(*n*-octadecyl acrylate) (PVP-*b*-PODA) by reversible addition-fragmentation chain transfer (RAFT) polymerization.

Synthesis of poly(*N*-vinyl pyrrolidone)-*block*-poly(*n*-octadecyl acrylate) (PVP-*b*-PODA): the amphiphilic block copolymer of PVP-*b*-PODA was synthesized by sequential reversible addition-fragmentation chain transfer (RAFT) radical polymerization technique of ODA and VP as shown in Scheme 1. First, poly(*n*-octadecyl acrylate) (PODA) was synthesized by RAFT radical polymerization using ACVA as an initiator and CTA. The number- and weight averaged molecular weight (*M*_n_ and *M*_w_) determined by ^1^H-NMR and size-exclusion chromatography (SEC) were 1.2 × 10^4^ and 1.3 × 10^4^, respectively. Sequentially, VP was polymerized by RAFT radical polymerization using the PODA as a macro-chain transfer agent. The number-averaged molecular weight (*M*_n_) of PVP-*b*-PODA determined by ^1^H-NMR and SEC were 3.4 × 10^4^ and 4.3 × 10^4^, respectively.

Preparation of PVP-*b*-PODA micelles: the obtained amphiphilic block copolymer was dissolved in nano-pure water at 1.0 mg/mL. The aqueous solution was homogenized with ultra-sonic homogenizer for 5 min. The aqueous solution was left at room temperature for over 24h.

Characterization: ^1^H-NMR measurements were performed by using a JEOL JNM ECP500 spectrometer (Tokyo, Japan). SEC measurements were performed by using a Shodex GPC K-804 column (eluent: chloroform, range of molar mass: 7000~300,000) combined with a JASCO (Tokyo, Japan) RI-4030 differential refractive index detector and a JASCO PU-2087 HPLC pump at a flow rate of 1 mL·min^−1^.

Small-angle X-ray scattering (SAXS): SAXS measurements were performed at the BL-40B2 and BL-03XU beamlines of SPring-8, Japan [16]. A 30 cm × 30 cm imaging plate (R-AXIS VII, Rigaku, Japan) was placed at a distance of 2 m from the sample position to cover a *q* range from 0.06 to 2.0 nm^−1^ at *λ* = 1.0 nm. Here, *q* = (4*π*/*λ*)sin(*θ*/2), where *θ* is the scattering angle and *λ* is the wavelength of the incident X-ray. A sample stage equipped with temperature controller was used. A sample solution was packed in a quartz capillary with a light path length of 2.0 mm (Hilgenberg GmbH, Malsfeld, Germany). The X-ray transmittance of the samples was measured with ion chambers located in front of and behind the sample. A sample was placed in the sample stage, annealed at a desired temperature for 10 min, and then exposed to the incident X-ray for 1min to obtain SAXS data with high signal-to-noise ratio. The two-dimensional SAXS images obtained with an imaging plate were converted into one dimensional scattering intensity versus *q* profiles by circular averaging. To obtain excess scattering intensity *I*(*q*) at each *q*, scattering from the background were subtracted from the raw scattering data after an appropriate correction of transmittance. The numerical analyses for SAXS data were carried out self-made programs on Igor 7 software (Portland, OR, USA) [17,18].

Dynamic light scattering (DLS): DLS measurements were performed to determine hydrodynamic radius (*R*_h_) of PVP-*b*-PODA micelle by using a DLS-7000 of Otsuka Electric Co., Ltd. (Tokyo, Japan) equipped with a temperature controller. A He-Ne laser (632.5 nm) was used as a light source. PVP-*b*-PODA micelle solution was placed at a desired temperature for 10 min, and then DLS measurements were performed at a scattering angle of 90°. Since the autocorrelation function decay monotonically at all temperature, *R*_h_s of PVP-*b*-PODA micelles were determined by the cumulant method.

2-dimensinal ^1^H-NMR spectroscopy: NOESY spectra of two-dimensional (2D) ^1^H-NMR for PVP-*b*-PODA micelles in D_2_O containing small amount of H_2_O were recorded by using a JNM-ECP500 spectrometer of JEOL (Tokyo, Japan). The scans were accumulated 64 times.

## 3. Results and Discussion

**Figure 1:**
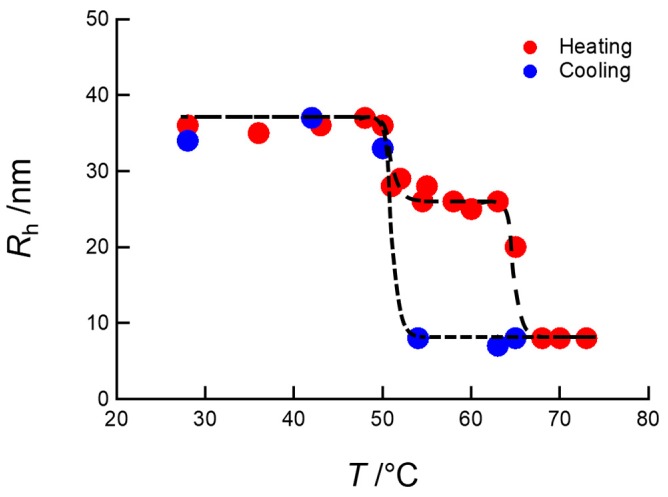
Change in hydrodynamic radius (*R*_h_) of PVP-*b*-PODA micelles with varying temperature.

Figure 1 shows temperature dependence of *R*_h_ of PVP-*b*-PODA micelles in heating and cooling processes obtained from DLS measurements. The *R*_h_ of PVP-*b*-PODA micelles undergoes two-step change in heating process, although it shows one-step change in cooling process. The temperature of first-step *R*_h_ decrement in heating process was consistent with *T*_m_ of PODA. In addition, the temperature of discontinuous *R*_h_ change in the cooling process agreed well with *T*_m_ of PODA. Therefore, it can be considered that the discontinuous *R*_h_ changes of PVP-*b*-PODA micelles around 50 °C in heating and cooling process are caused owing to melting and crystallization of PODA, respectively. On the other hand, the second-step of discontinuous *R*_h_ decrement observed only in heating process is observed around 65 °C in spite of absence of phase transition around 65 °C. Therefore, it is considered that PVP-*b*-PODA micelles take transient state from *T*_m_ of PODA to 65 °C in heating process. In order to confirm the structural change of PVP-*b*-PODA micelles during heating process, SAXS measurements were performed.

**Figure 2:**
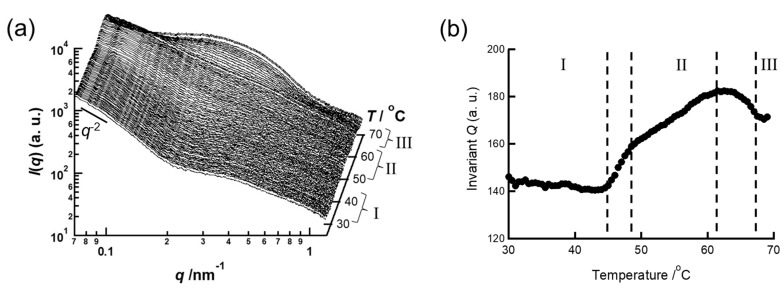
Changes in small-angle X-ray scattering (SAXS) profiles (**a**) and Invarian *Q* (**b**) of PVP-*b*-PODA micelles with elevating temperature.

Figure 2a,b show changes in the SAXS curves and the invariant *Q* of the PVP-*b*-PODA micelles, respectively, with elevating temperature. Here, invariant *Q* is defined by the following equation:

(1) $$\mathrm{invariant}\;Q=\;\frac{1}{2\pi }\overset{\infty }{\underset{0}{\int }}q^{2}I\left(q\right)dq$$

where *I*(*q*) is scattering density at *q* and *q* is the magnitude of scattering vector defined by (4*π*/*λ*)sin(2*θ*). Here, *λ* is the wave length of incident X-ray and 2*θ* is scattering angle. The SAXS curves and invariant *Q* are classified into three stages indicating I (room temperature ~45 °C), II (48~62 °C), and III (>67 °C) in Figure 2a. The SAXS curves are changed as the stage changes. This means the transitions of stages are occurred due to transformation of PVP-*b*-PODA micelles. Since the transition temperatures from stage I to II and stage II to III correspond to those of discontinuous *R*_h_ decrement shown in Figure 1, the two-step *R*_h_ change of PVP-*b*-PODA micelles in heating process is related to transformation of micelles. The SAXS curves in stage I shows *q*^−2^ dependence of *I*(*q*) in *q* < 0.1 nm^−1^. This means PVP-*b*-PODA micelles form disk-like shape below 45 °C. The formation of disk-like shape should be caused by crystallization of PODA in the hydrophobic core of PVP-*b*-PODA micelles. As mentioned above, the temperature of first step change in heating process is caused due to melting of PODA. Therefore, the melting of PODA in the hydrophobic core causes transformation of PVP-*b*-PODA micelles. In the SAXS curves in stage II, a strong q dependence of *I*(*q*) in *q* < 0.2 nm^−1^ was observed. This suggests that PVP-*b*-PODA micelles form an anisotropic shape in stage II. On the contrary, in SAXS curves in stage III, *I*(*q*) in *q* < 0.2 nm^−1^ did not show *q* dependence. Therefore, PVP-*b*-PODA micelles should form spherical shape in stage III. In order to elucidate the shape change of PVP-*b*-PODA micelles, fitting analyses for these SAXS curves were carried out by using theoretical scattering functions for model particles.

**Figure 3:**
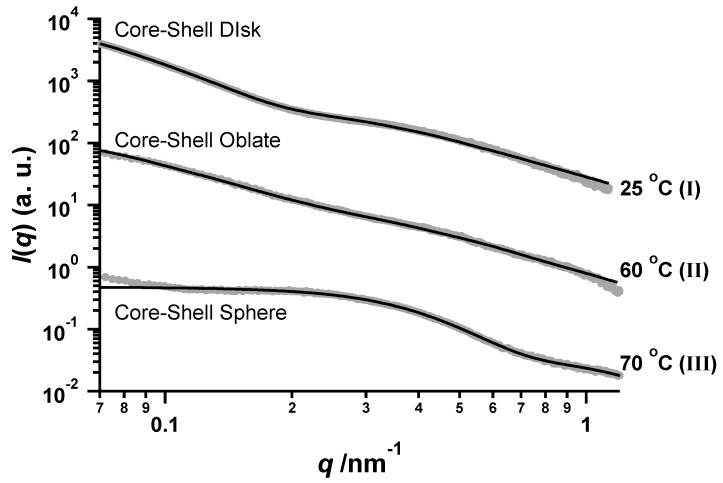
Fitting analyses of SAXS profiles of PVP-*b*-PODA micelles at 25, 60, and 70 °C by using core-shell disk, oblate, and sphere, respectively.

Figure 3 shows the results of fitting analyses for typical SAXS curves in stages I, II and III. Table 1 summarizes the parameters yielding the best fitted results and the solid lines in Figure 3 are the best fitted results. For the SAXS curve at 25 °C, the theoretical scattering curve calculated for a core-shell disk given by the following equation [12,19] agreed well with the experimental SAXS curve:

(2) $$I\left(q\right)\propto \int_{0}^{\pi }{\left[\left(\rho_{C}-\rho_{S}\right)V_{C}F_{C}\left(q\right)+\left(\rho_{S}-\rho_{0}\right)V_{S}F_{S}\left(q\right)\right]}^{2}\sin\beta d\beta$$

(3) $$F_{i}\left(q\right)=\left[\sin\left(\frac{qt_{i}}{2}\cos\beta \right)/\left(\frac{qt_{i}}{2}\cos\beta \right)\right]\frac{2J_{1}\left(qR\sin\beta \right)}{qR\sin\beta }$$

Here, *F*_i_(*q*) the scattering amplitude of i (I = C: core, S: shell, 0: solvent). The *R*, *ρ*_i_, *V*_i_ and *t*_i_ are disk radius, the electron density, volume, and thickness of i (I = C: core, S:overall, 0:solvent), respectively. *J*_1_ denotes the Bessel function of the first kind and of the order 1, and *β* is the angle between the axis of symmetry of particle and the scattering vector *q*. By using *t*_C_/2 = 2.8 nm, we obtained the best fitted result. On the other hand, for the SAXS profile at 60 °C, the scattering curve calculated for ellipsoidal oblate given by the following equation is in good agreement:

(4) $$F_{i}\left(q\right)=3\frac{\sin\left(qr_{i}\right)-qr_{i}\cos\left(qr_{i}\right)}{{\left(qr_{i}\right)}^{3}}$$

(5) $$r_{i}={\left[\left(a_{i}{}^{2}\sin^{2}\theta +b_{i}{}^{2}\cos^{2}\theta \right)\sin^{2}\varphi +a_{i}{}^{2}\cos^{2}\varphi \right]}^{1/2}$$

Here, *a*_i_ and *b*_i_ are short and long semi-axis of i. When *a*_i_ = *b*_i_, Equation (4) corresponds to the scattering function of spherical particle. In this calculation, we used *a*_C_ = 3.1 nm, which is slightly longer than the *t*_C_/2. In addition, the electron density of shell at 60 °C is the same as that at 25 °C, although the electron density of hydrophobic core is drastically decreased due to melting of PODA. Furthermore, at 70 °C, the experimental SAXS curve agreed well with the theoretical scattering curve for core-shell sphere with *r*_C_ = 2.0 nm and *r*_S_ = 5.5. Here, the electron density of core-shell at 70 °C is lower than that at 60 °C. Therefore, PVP-*b*-PODA micelles transform from disk to sphere via ellipsoidal oblate between *T*_m_ of PODA and 65 °C, although the shape of PVP-*b*-PODA micelles is directly changed from sphere to disk owing to crystallization of PODA in the cooling process. It seems that the PVP-*b*-PODA micelles memorize the shape formed at room temperature above *T*_m_ of PODA. In order to maintain the anisotropy of shape against transformation to thermodynamically stable shape, it is necessary for the PVP-*b*-PODA micelles to have a trick like a cross-liking in corona layer. It has been known that the PVP strongly hydrates at ambient temperature and dehydrates around 70 °C. If pseudo network in corona layer of PVP-*b*-PODA micelles is formed owing to robust hydration like a cross-linking, the shape of PVP-*b*-PODA micelles formed below *T*_m_ of PODA should be memorized above the *T*_m_. Actually, we confirmed that poly(ethylene glycol)-*block*-PODA (PEG-*b*-PODA) micelles, in which hydration of PEG is much weaker than that of PVP, shows one-step transformation from disk to sphere at *T*_m_ of PODA in heating process (see Figure S1 in supporting information). Therefore, it is considered that the shape change from disk to sphere via ellipsoidal oblate during the heating process is related to the dehydration of PVP in the corona layer. In order to investigate the hydration of PVP in the corona layer, two-dimensional ^1^H-NMR measurement was performed on the PVP-*b*-PODA micelles in deuterated water containing small amount of H_2_O.

**Figure 4:**
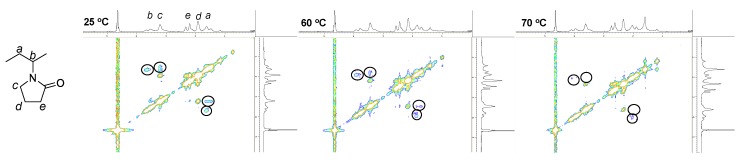
Two-dimensional (2D) ^1^H-NMR (NOESY) spectra of PVP-*b*-PODA micelles measured at 25, 60, and 70 °C.

Figure 4 shows two-dimensional ^1^H-NMR NOESY spectra. The signals indicated with circles in Figure 4 indicate that protons of H_2_O and PVP (denoted as b and c in Figure 4) are spatially close each other. They appear when PVP chains are strongly hydrated [13,14]. They are clearly observed at 25 and 60 °C, and disappear at 70 °C. This indicates that PVP chains in the corona layer are still hydrated above *T*_m_ of PODA, and dehydrated at 70 °C. The temperature at which PVP chains were dehydrated corresponds to that at which the PVP-*b*-PODA micelles transform from ellipsoidal oblate to sphere. Therefore, it is considered that the shape memory phenomenon in PVP-*b*-PODA micelles in the heating process is caused by the robust hydration of PVP in corona layer to form pseudo network.

**Figure 5:**
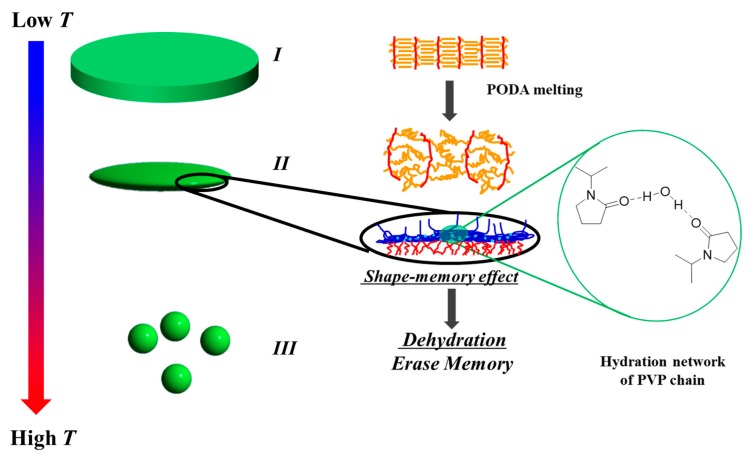
Schematic illustration of molecular mechanism of two-step temperature responsiveness of PVP-*b*-PODA micelles in heating process.

By summarizing the results as mentioned above, the molecular mechanism of two-step shape change of PVP-*b*-PODA micelles in heating process is schematically illustrated in Figure 5. The PVP-*b*-PODA forms disk-like micelles below *T*_m_ of PODA, because crystallization of PODA in the hydrophobic core creates a flat interface between hydrophobic core and hydrated corona. When the temperature rises above *T*_m_ of PODA, the PVP-*b*-PODA micelles tend to change into a spherical shape according to the volume fraction of core and corona. However, the robustly hydrated network in PVP corona prevents drastic change in the curvature of the interface, so that the PVP-*b*-PODA micelles become ellipsoidal oblate in which disk-like shape below *T*_m_ of PODA is memorized. Finally, when the PVP-*b*-PODA micelles are heated above 65 °C, the memory of disk-like shape is erased due to dehydration of PVP and the PVP-*b*-PODA micelles change to spherical shape. On the contrary, during the cooling process, the shape of PVP-*b*-PODA micelles is simply changed from sphere to disk as shown in Figure 1. This suggests that the hydration network in the PVP corona layer can overcome the change from the flat interface to the curved interface triggered by the melting of PODA, although the change from curved interface to flat interface triggered by crystallization of PODA is superior to effect of maintaining the shape of micelle by hydration network in PVP corona. The shape memory effect by hydration network in the corona layer on polymer micelles has not been reported and it can be expected to give a design guideline for creation novel stimuli-responsive polymer micelles.

## 4. Conclusions

In this study, the two-step temperature responsiveness of PVP-*b*-PODA micelles in heating process was found by DLS and SAXS. SAXS revealed that the temperature responsiveness of PVP-*b*-PODA micelles were attributed to shape changes from disk to ellipsoidal oblate and form ellipsoidal oblate to sphere. The first-step shape change was triggered by melting of PODA in hydrophobic core and the second-step was caused by dehydration of PVP in corona layer. Furthermore, it was suggested that the appearance of the ellipsoidal oblate between *T*_m_ of PODA and 65 °C was caused due to shape memory effect by formation of robust hydration network of PVP chains in corona layer.

## Figures and Tables

**Table 1:** Models and adjustable parameters used in fitting analyses for SAXS profiles of PVP-*b*-PODA micelles.

Temperature	Shape	Size	*ρ*_C_/e^−^ nm^−3^	*ρ*_S_/e^−^ nm^−3^
25 °C	Core-Shell Disk	*t*_C_/2 = 2.8 nm, *t*_S_/2 = 5.9 nm	336	335.3
60 °C	Core-Shell Oblate	*a* = 3.1 nm, *a*_S_ = 6.1 nm *b* = 67.5 nm, *b*_S_ = 70.5 nm	332	335.3
70 °C	Core-Shell Sphere		332	334.5

*a*_S_: short semi-axis of overall micelle. *b*_S_: long semi-axis of overall micelle.

## Supplementary Materials

The supplementary materials are available online at http://www.mdpi.com/2073-4360/11/2/382/s1.
