# Supplementary material for: Effect of Hydration in Corona Layer on Structural Change of Thermo-Responsive Polymer Micelles
Source: Polymers (Basel). 2019 Feb 22;11(2):382. doi: 10.3390/polym11020382 (PMC6419195; doi:10.3390/polym11020382)
Supplement: Supplementary file 1 [file polymers-11-00382-s001.pdf]

Supporting information for

Effect of Hydration in Corona Layer on Structural Change of Thermo-responsive Polymer Micelles

Yusuke Akino, Kosuke Morimoto, Kengo Tsuboi, Satoshi Kanazawa and Isamu Akiba*

Department of Chemistry and Biochemistry, The University of Kitakyushu, 1-1 Hibikino, Wakamatsu, Kitakyushu 8080135, Japan; akiba@kitakyu-u.ac.jp

* Correspondence: akiba@kitakyu-u.ac.jp; Tel.: +81-93-695-3295

Contents

Small-angle X-ray scattering (SAXS) from poly(ethylene glycol)-block-poly(octadecyl acrylate) (PEG-b-PODA) micelles at 25 and 50 ºC.

SAXS from PEG-*b*-PODA micelles.

In order to compare temperature responsiveness of PODA containing polymer micelles, change in SAXS profiles of micelles consisting of poly(ethylene glycol)-*block*-poly(octadecyl acrylate) (PEG-*b*-PODA) with elevating temperature was investigated. Here, number- and weight-averaged molecular weight (M_n and M_w)of PEG block are 3.0 x 10^4 and 3.5 x 10^4, respectively, and Mn and Mw of PODA were 1.3 x 10^4 and 1.5 x 10^4, respectively.

Figure S1 shows SAXS profiles from PEG-*b*-PODA micelles at 25 ºC (below melting temperature (T_m) of PODA) and 60 ºC (above T_m of PODA). SAXS curve measured at 25 ºC shows q^{-2} dependence of $I(q)$ in q < 0.2 nm^{-1}. This means PEG-*b*-PODA micelles form disk-like shape. Actually, the experimental SAXS curve of PEG-*b*-PODA at 25 ºC well agree with the theoretical scattering curve calculated for core-shell disk particle shown as solid line in Figure S1. On the contrary, in the SAXS curve measured at 50 ºC, q^{-2} dependence of $I(q)$ is disappeared and $I(q)$ does not show q dependence in q < 0.2 nm^{-1}. Therefore, at 50 ºC, PEG-*b*-PODA micelles form spherical shape. Therefore, in the case of PEG-*b*-PODA micelles, melting of PODA simply causes transformation from disk-like to spherical shape, although two-step transformation from disk to sphere *via* ellipsoidal oblate is observed in PVP-*b*-PODA micelles.

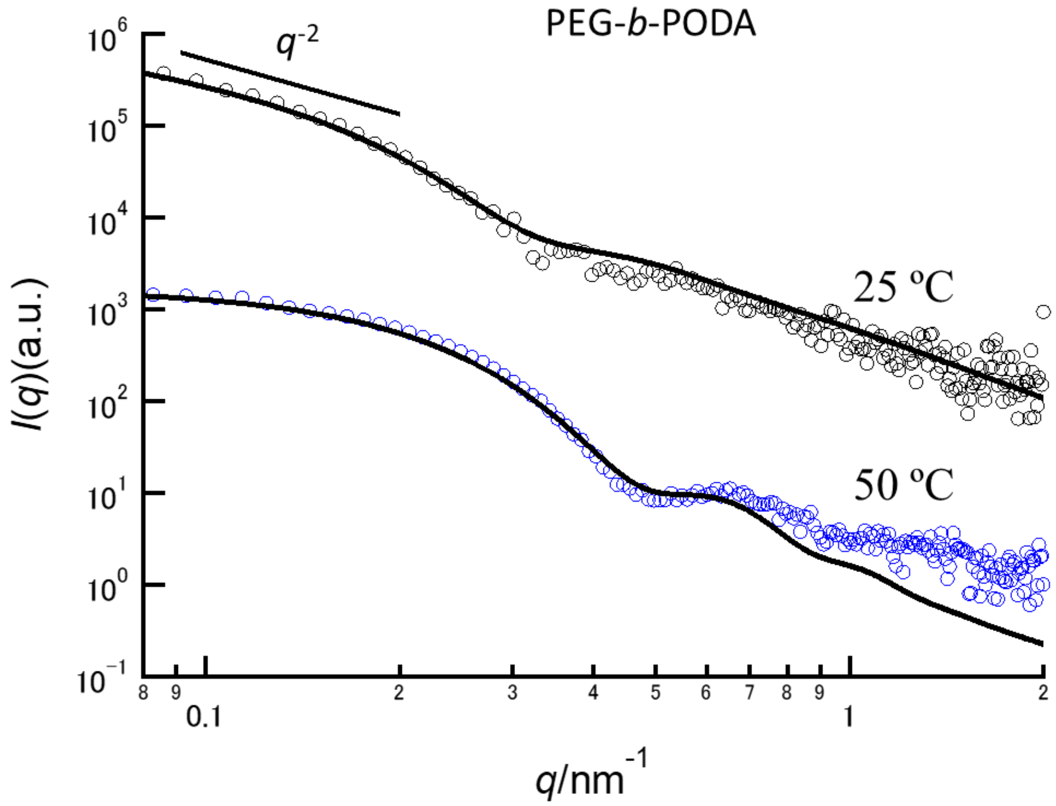

Figure S1. SAXS profiles of PEG-*b*-PODA micelles in aqueous solution at 25 ºC (below T_m of PODA) and 50 ºC (above T_m of PODA).
